# “What you feel under your hands”: exploring professionals’ perspective of somatic dysfunction in osteopathic clinical practice—a qualitative study

**DOI:** 10.1186/s12998-022-00444-2

**Published:** 2022-08-31

**Authors:** Lorenzo Arcuri, Giacomo Consorti, Marco Tramontano, Marco Petracca, Jorge Eduardo Esteves, Christian Lunghi

**Affiliations:** 1 Malta ICOM Educational, Gżira, Malta; 2 Education Department of Osteopathy, Istituto Superiore di Osteopatia, Milan, Italy; 3 Fondazione Santa Lucia IRCCS, Rome, Italy; 4 Centre Pour L’Etude, la Recherche et la Diffusion Ostéopathiques, Rome, Italy; 5 Osteopathic Service, Osteobimbo Paediatric Clinic, Rome, Italy; 6 Foundation COME Collaboration, Pescara, Italy

**Keywords:** Manipulation, Osteopathic, Somatic dysfunction, Educational models, Theoretical models

## Abstract

**Background:**

Despite controversy regarding its validity and clinical usefulness, manual examination findings still have an important role for manipulative therapies. As an example, somatic dysfunction (SD) remains central to osteopathic practice.This study aims to explore the experienced osteopaths' attitudes concerning SD and its role in osteopathic practice. This qualitative research could contribute to building a consistent paradigm for manual intervention in all musculoskeletal manipulations.

**Methods:**

A thematic analysis with grounded theory elements was used. Data were collected through semi-structured interviews carried out between February and April 2021. A purposive sample of twenty professional osteopaths with past experience in osteopathic care was chosen to reflect the phenomenon's variety. The data analysis was done inductively and in tandem with the recruiting to keep track of data saturation.

**Results:**

Eleven osteopaths participated in the study. Three main themes emerged from the data analysis: (1) SD as a safe tissue-touch-based communication tool between operator and person complex adaptive health system; (2) The treatment of SD is shareable between osteopaths, other health professionals, and the patients involved in the therapeutic pathway improving body awareness and health; (3) The development of the SD concept in research and practice to better clarify osteopathic profession identity and definition.

**Conclusions:**

A panel of expert osteopaths consider the concept of SD as a valuable tool integrated into the osteopathic evaluation and treatment process. The shared concept and clinical application of SD is informed by person-centered care concepts and from the fields of neuroscience, cognitive and complexity science. Our study reports a common need among osteopaths to develop an evidence-based framework of SD to allow the best development of the osteopathic profession.

**Supplementary Information:**

The online version contains supplementary material available at 10.1186/s12998-022-00444-2.

## Introduction

Manual and musculoskeletal therapists (e.g. physical therapists, chiropractors and osteopaths) apply various hands-on\hands-off or through equipment interventions on the whole body framework to meet the patients’ need improving health,circulation, relieve fatigue and promote healing [1].

Nowadays, there is an emergent debate about the reconceptualization of the value of manual examination and palpatory findings generally used by manual therapists [2], chiropractors [3], and osteopaths [4].

Articular or neural manipulative techniques have been linked to biomechanical rationale effects, even if their association with clinical results is controversial [5, 6]. What we do know is that manual therapists use biomechanical rationale to explain their clinical practice [5, 6]. Conversely, we also know that research only supports temporary movement improvement effects [5, 6]. Moreover, it is known that positional changes are not well documented [5, 6]. Consequently, biomechanical palpatory evaluation has shown poor reliability [5, 6]. However, manual techniques applied in remote areas results in improvements of musculoskeletal signs and symptoms [7].

In the field of manual therapy a current model suggests that the mechanical force from a manual therapy intervention results in systemic neurophysiological responses leading to pain inhibition [5, 6, 8]. What we don't know are the attitudes, the beliefs of contemporary experienced manual therapists about the role and applications of palpatory findings in patient-centered care, and the adherence of their perspective to current knowledge.

It matters because nowadays it is well known that musculoskeletal pain conditions are a result of a complex interaction of biological, and psychological factors and cannot be resolved by addressing structural dysfunction/impairment alone. There is a need for manual therapies, such as osteopathy and chiropractors to move beyond structural impairments and achieve a renovated shared paradigm for musculoskeletal pain care. There is an existing empirical and theoretical gap in the literature that needs to be better articulated to serve as rational for future study and creates a springboard for a renovated model underlying palpatory finding and their use in manual therapies. There are recommendations to advance the study of palpatory findings and conceptual models for manual therapies [10] that include results of qualitative research to generate a theoretical construct. Qualitative findings will help to better understand practitioners' assumptions; obtain input on consistency, plausibility, generalisability, relevance, and expected applicability from experts, practitioners, and patients. The effects of a renovated model might consider the growing understanding of a more person-centered approach in the use of palpatory findings: when integrated with multidimensional patient profiles and person-centered approaches, the selective and informed use of these palpatory findings still has an important role to play in manual therapy clinical practice [2].

Osteopathic care uses manual techniques to support individual psychophysiological adaptation [9]. The treatment has been described to be focused on somatic dysfunction (SD) [9–12], which could be defined as an altered function of the body's framework system components. Although in the USA, there is a general consensus on the use of SD in the Osteopathic Manipulative Treatment (OMT), in Euro-Australasia there is a discussion about the usefulness of the concept for contemporary osteopathic care [13].

The international osteopathic community is now facing a growing debate on the role of SD in osteopathic practice and professional identity [13–20]. There is a need to critically examine the heritage that osteopathy carries, in order to build a strong osteopathic professional identity and clinical practice that clearly differentiates from other professions [21, 22]. The entire osteopathic community, by the fear of losing osteopathic identity, did not detach from the past beliefs and did not allow the profession to grow and develop [14, 16, 18]. There is a need to clarify the core osteopathic body of knowledge (including the concept of SD) and define the profession's contribution to managing health needs and the quality of health services [20]. The implication for the concept of SD involves different stakeholders, both clinicians, researchers, and educators. To promote a more robust professional identity and the legitimacy of osteopathy as a mainstream healthcare field, a global standard model for SD is required.

Several studies proposed theoretical frameworks for SD [11, 23–28]. Others like Licciardone et al., 2014 reported SD use during ambulatory medical care visits [29]. However, only a few authors reported the osteopaths' direct thoughts on SD in clinical practice [14, 19, 20, 30–32]. Moreover, a recently published scoping review highlighted a gap between osteopathic clinical practice and the osteopathic methods reported in the literature concerning the use of SD [28]. The results of the review highlight that on the one hand there is an underlying assumption that the notion of a 'somatic dysfunction' exists as a clinical entity. On the other hand there is no universal agreement that SD exists or underpins the practice of osteopaths worldwide.To the best of our knowledge, there are no studies that qualitatively investigate osteopaths’ perspectives on the role of SD in patient care.

Considering the growing debate about the value of SD in the osteopathic profession, there is a need to investigate the preferences of expert osteopaths in the clinical application of palpatory findings.

We hypothesize that a better knowledge of attitudes and preferences concerning palpatory findings application in clinical osteopathic practice could clarify to the research and practice community the effective application of SD in the osteopathic clinical setting and may represent a starting point to develop a common framework for manual assessment in musculoskeletal manipulations. Indeed, a recent call is launched for qualitative research on the attitudes and beliefs of osteopaths regarding the role of SD in clinical practice [32].

For these reasons, this qualitative study aims to explore the experienced osteopaths' attitudes concerning SD and its role in osteopathic practice.

## Materials and methods

A qualitative examination of experienced osteopaths’ perspectives could improve the interpretative model on the use of SD. Considering the value of the practitioners’ experience would help structure a common framework for the palpatory findings used in manual therapies. Indeed, qualitative research evidence has great significance for evidence-based, person-centered treatment [33]. Qualitative study findings, adding value to quantitative research results, can provide an adequate understanding of the complex interaction between the healthcare system and the setting in which persons, communities, and populations are cared for, according to policy and decision-making.

According to the implications for conducting a qualitative descriptive study [34, 35] the authors chose thematic analysis as the major framework since the current study's issue necessitates evaluating narrative resources of life experiences and a theme presentation of the pre-given essences and structures of lived experiences [34, 36, 37]. Furthermore, grounded theory elements were implemented during the coding phase to better seize the meaning of the data [38]. Thematic analysis is defined as "a strategy for detecting, analyzing, and reporting patterns (themes) within data" as an independent qualitative descriptive approach [35]. The aim and concentration of thematic analysis are to analyze narrative materials of life stories and give a thematic description of the pre-given essences and structures of lived experiences [34, 35]. The philosophical background is a constructivist perspective [34, 35]. The analysis process provides a description and interpretation, both inductive and deductive, emphasizing context, integration of manifest and latent contents, drawing a thematic map, and a non-linear analysis process [34, 35].

**Fig. 1:**
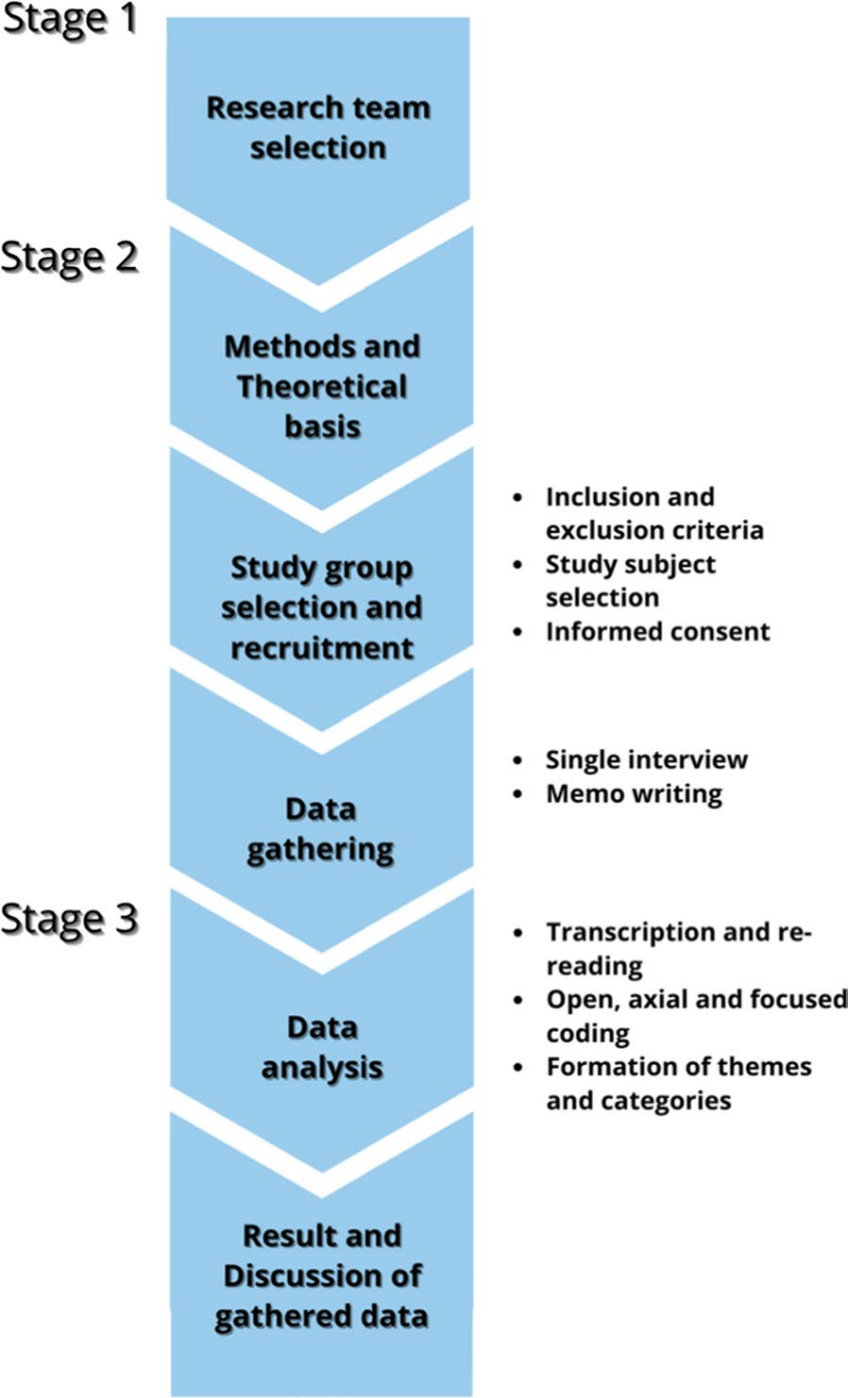
Study development

**Fig. 2:**
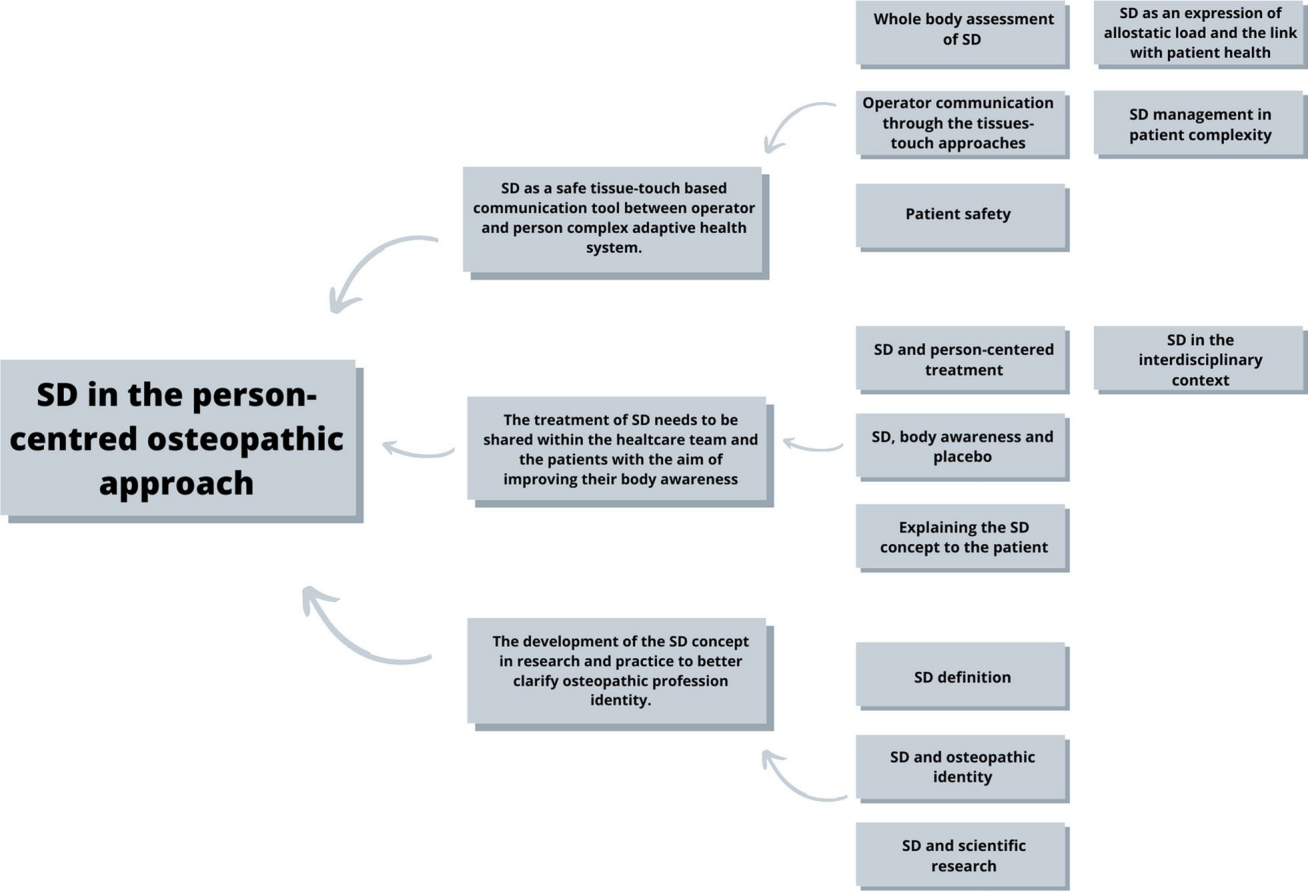
Themes and categories

Data were collected through semi-structured interviews developed through a consensus statement between two researchers (L.A., C.L.) [34–36]. Questions and prompts of the interview's draft were then reviewed by two other researchers with relevant osteopathic clinical and methodological expertise (GC, MT). Qualitative data were then processed according to recommendations for thematic analysis [34]. The good methodology and development of the study (Fig. 1), was checked by referring to the Consolidated Criteria for Reporting Qualitative Research (COREQ) checklist [39]. The protocol was designed by four osteopaths with relevant clinical and methodological expertise (CL, JE, GC, MT) and was registered with protocol number 10.17605/OSF.IO/38AVR on Open Science Framework Registry. This study was approved by the Local Ethics Committee of Malta ICOM Higher Institution (4/02/2020N. AL000296MIFT). The quality criteria used in the present study to ensure trustworthiness is based on credibility, transferability, dependability, and confirmability [34–37]. Credibility was satisfied by checking and correction of interview transcripts by participants; by implementing the simultaneity of the interview, transcription, re-reading, and category\themes formation phases; by the creation of memo writing [34–37]. The principal researcher (L.A.) and an experienced osteopath in qualitative research (C.L.) have done category and theme generation to validate the process [34–37]. Then compared with another experienced qualitative researcher (G.C.). Furthermore, credibility was ensured by member checking [34–37]. Participants are given data or outcomes to check for accuracy and resemblance to their own experiences. The audio recordings were transcribed and then were sent back to the respondents in order to edit and/or confirm them. Respondent validation enables participants to add comments which are then searched for confirmation or disconfirming resonance with the analyzed study data, enhancing the credibility of the results [34–37]. To favor the single participants' comprehension and see the bigger picture with data gathered from multiple participants the Synthesized Member Checking method was implemented [40]. The sequenced five-step process is as follows: prepare a summary from emerging themes, check the participants' eligibility to receive the report, send out the Synthesized Member Checking report with a cover letter to ask participants to read, comment, and return, then gather responses and added data, and integrate findings. Transferability was met by group selection heterogeneity and data saturation [34–37]. Furthermore, transferability was confirmed by obtaining thick descriptions from participants during the interviews, and sharing and debating emergent concepts and theories with other osteopathic practitioners who were not participating in the study [41]. To fulfill dependability, and confirmability, the thematic analysis results were discussed and shared by the principal researcher (L.A.) with all other authors, experts in qualitative research (C.L., G.C.,J.E.) [34–37]. Confirmability was achieved through a sharing process in which the researchers verified that their findings were data-driven [34–37]. Moreover, the audit trail technique was used to ensure both dependability and confirmability: the entire process was supervised by the researcher least involved in the data gathering (M.T.) [34–37]. The process of constructing categories and themes is demonstrated and can be traced through its schematization (Fig. 2). To address possible researchers’ biases towards the topic which could have potentially interfered with the data analysis, the authors underwent a reflective process. Schwandt defines reflexivity as: (a) “the process of critical self-reflection on one’s biases, theoretical predispositions, preferences”; (b) an acknowledgement that “the enquirer is part of the setting, context and social phenomenon they seeks to understand”; and (c) “a means for critically inspecting the entire research process” [42]. The reflective process was carried out prior to the data gathering and consisted in a group meeting where researchers’ were invited to discuss the topic of the interviews. All personal opinions were written on notes and the notes were used to check possible influences during the confirmability process [43].

### Sample

#### Study group selection and recruitment

The authors selected a purposeful sampling to identify information-rich cases relating to the phenomenon of interest [44]. Moreover, the principal researcher requested referrals from other participants to enrich the selection with experienced osteopaths with specific knowledge about SD. To guide the adequate sample size of the present qualitative study we implemented the concept of “information power”: the more information the sample holds, relevant for the actual study, the lower the number of participants is needed [45]. To achieve a suitable size of a sample with sufficient information power the authors considered the aim of the study, the sample specificity, the use of established theory, the quality of dialogue during the interview, and the selection of analysis strategy [45].

Twenty expert osteopaths [46] were purposely selected among Registro degli Osteopati d’Italia (ROI) [47] and contacted [36] via email. The email contained all the information regarding the study aim and methodology, specifying the role of participants and asking for their interest in participating in the interviews. Potential participants contacted by the principal researcher (L.A.) by email were requested to confirm meeting the inclusion criteria. The principal researcher (L.A.) screened for eligibility. Moreover, informed consent for the use of personal data and the audio recording of the interviews was provided as an attachment (to be signed and returned to the author before the interview).

#### Inclusion and exclusion criteria

Inclusion criteria were: expert osteopaths who had at least 10,000 h of clinical and academic practice [36, 46]. Participants who have a personal relationship with the interviewer were excluded.

To achieve a highly specific combination of participants for the study aim, with exhaustive knowledge on the updated models and theories concerning SD, potential participants who did not show experience in the academic or research field were excluded. The size of the study group was dependent on the saturation of the collected data [36, 37].

### Data gathering

A single semi-structured interview was conducted with each study participant, who was engaged by the investigators in an inductive, in-depth, recursive, and deep plowing conversation to better express their clinical experience regarding SD [34, 36, 37]. The interviews, consisting of 9 main questions with several other sub-questions [See Additional file 1], were conducted on the Zoom online platform. The authors selected Zoom web platform since there is an agreement among researchers that video conferencing is a useful method for conducting qualitative interviews. There are available research findings identifying Zoom as a preferred method compared to in-person interviews, telephone, or other video conferencing platforms [48].

Such technologies mimic the characteristics of face-to-face interviews (i.e., the ability to convey and respond to verbal and nonverbal cues) while also offering distinct benefits, challenges, and considerations such as ease, simplicity, and user-friendliness [49]. Moreover, using a web video conferencing platform allow the principal researcher (L.A.) to better engage in reflexivity: writing notes about participants’ comments and researcher’s thoughts during the interview; memoring as soon as possible after an interview (having the possibility to review the recording), and developing and continually editing the researcher’s subjectivity statement [50].

Each meeting was audio recorded in order to allow transcription and data analysis [34, 36, 37, 39]. The interviews were conducted by the principal researcher (L.A.), a male osteopath, with BSc credential, and with curricular training on the implementation of qualitative research projects. Authors planned with participants an interview between 30 and 60 min. During the interview, the interviewer kept a memo writing in order to make the subsequent formation of the categories more adherent. Before each interview, participants signed an informed consent form, which explained the characteristics of the study and how personal data would be used. Throughout the duration of the study, participants were guaranteed anonymity and were kept up-to-date on the development of the study. The study was conducted in accordance with the Declaration of Helsinki [51].

### Thematic analysis

The interviews were carried out between February and April 2021 by the principal researcher (L.A.). In the same period verbatim transcription, re-reading of the texts, and the first idealization of the categories were carried out by the author of the interviews (L.A.) and another osteopath expert in qualitative research (C.L.). The interview transcriptions were emailed to each interviewee, who was given the opportunity to check, correct, or edit the text [34, 36, 37]. Once the corrections were received from the participants (specifically, participants 4, 6, 7 sent corrections regarding the syntax of the text), the authors re-read the texts for the third time. Next, thematic analysis of the texts was conducted individually by two authors (by L.A. and C.L), and through an inductive process were generated categories by the same authors through open, axial, focused line-by-line coding as described by grounded theory framework [38], comparing them to memo writing [34, 36, 37]. Subsequently, results were shared between the first two authors and another researcher (G.C.) to meet a better consensus on data.

Thematic analysis as an independent qualitative descriptive approach is mainly described as “a method for identifying, analyzing and reporting patterns (themes) within data” [34, 35]. The interviews were declared completed when all the fields of interest were saturated. The strategy given by Guest et al. (2020) was employed to accomplish the saturation process, defining a stop if the new information collected falls below 5% [52]. Once the creation of the categories was completed, the authors re-read texts and the corresponding categories in order to outline the themes that emerged. After comparing the categories, it was possible to structure the final themes. Authors had no prior knowledge of the participants and recognized his role in interpreting the analyzes.

## Results

Twenty experienced osteopaths were contacted by email, eighteen responded to the first email, showing interest in participating in the study. Seven out of eighteen dropped out from the interview for work reasons; eleven experienced osteopaths participated in the study. Demographic characteristics showed in general heterogeneity of the study group (Table 1).

**Table 1:** Demographic characteristics (n = 11)

Age (mean ± SDV)	43.6 ± 9.4
Years of practice (mean ± SDV)	15.7 ± 7.6
Years of academic teaching and tutoring	
(mean ± SDV)	11.0 ± 7.8
Gender (n)	
Male	10
Female	1
Topography (n; %)	
North Italy	5; 45.5
Center Italy	4; 36.4
South Italy	2; 18.2
Training in osteopathy (n; %)	
Diploma in osteopathy (Italy, France)	11; 100
Bachelor in science of osteopathy (United Kingdom, Germany)	4; 36.4
Master in science of osteopathy (United Kingdom)	1; 9.1
Previous training (n; %)	
Degree in physiotherapy	5; 45.5
Degree in sport sciences	2; 18.2

n = Number; SDV = Standard deviation; % = percentage

Data saturation was reached by the ninth participant and was confirmed with a further two interviews (Table 2) [52]. Interviews were therefore carried out as shown in Table 2. The average time of the interviews rose to 37.5 min (Standard deviation: 10.5).

**Table 2:** Interviews' saturation process

Interview number	1	2	3	4	5	6	7	8	9	10	11	Tot
Base themes	12	8	4	3	2							29
New themes in run						3	1	1	0	0	0	5
% change over base (threshold of ≤ 5)						13.7		3.4				

From the thematic analysis of the interviews, three main themes emerged:

1. SD as a safe tissue-touch based communication tool between operator and person complex adaptive health system;
2. The treatment of SD needs to be shared within the healthcare team and the patients with the aim of improving their body awareness;
3. The development of the SD concept in research and practice to better clarify osteopathic profession identity.

The mentioned themes compose an overarching theme representing participants' uses for SD as a part of the person-centered osteopathic approach. Figure 2 graphically represents the three main themes concepts applied in osteopathic clinical practice. The most representative participants' verbatim quotes are reported in the following paragraphs. All the most relevant participants’ quotes are reported in Additional file 2.

Theme 1: SD as a safe tissue-touch based communication tool between operator and person complex adaptive health system [see Additional file 2]

In general, all participants reported integrating SD into the overall assessment of the patient. SD, in this case, is used as one of the elements to interpret the person's needs without being the main focus of the osteopathic rationale (in which the whole person is at the center). The SD is then integrated with other elements such as adaptive capability and allostatic load (AL) index, expression of daily functions of the patient, sensations, and perceptions of the patient, and progression of symptoms.

SDs impact on the subject's adaptability…then it can cause symptoms at that point. (P1)

Participants (55%) referred to the palpable clinical signs of tissue texture alteration, positional asymmetry, altered range of motion, tenderness, classically indicated by the TART acronym (P2, P3, P4, P7, P9, P10); Some participants considered movement as the main parameter (P5, P6, P11). In order to deepen the analysis of the SD and to understand its relationships with the other parts of the body, some participants declared to rely on specific tests of palpation and relation between structure and function (P2, P4, P6, P7, P9, P10). The palpatory findings highlighted in the first assessment phase should be integrated into person-centered clinical reasoning before having values in the shared decision-making and treatment process. Supposing the SD under examination does not evoke any patient perception and responsiveness in terms of improving body functions, familiar symptoms, or daily movement; in that case, it would be re-assessed and eventually considered during the progression of osteopathic treatment (P1, P2, P3, P4, P5, P6, P7, P10).

SD can be somehow parameterized or otherwise quantified through parameters defined by the TART acronym. (P4).

I only take SD into account if clinically reflects the expression of an altered functioning of the patient, otherwise it is negligible … it must always be placed in a context of globality. (P4).

The parameter that osteopaths objectively use the most is mobility and movement especially in the first degrees. (P6).

Participants reported the importance of touch and palpation in both assessment and treatment phases. In particular, they claimed to establish a non-verbal communication based on contact: metaphorically speaking, they refer to establishing a dialogue between the patient's tissues, the practitioner's hands, and the patient itself. Such a participatory approach is based on improving patient body awareness to better understand the patient's needs through an osteopath-patient dyadic mutual influence (P2, P4, P5, P7, P10, P11).

The best technique for a specific SD you can feel it in the moment. It can't be categorized. It depends a lot on what you feel under your hands. (P7).

In assessment and treatment there is a constant exchange of information between the hands and the body that tells you the type of approach to be applied, the force to be applied and the direction to be taken… the ability to have a continuous dialogue with the tissue then translates into the effectiveness of the treatment. (P11).

During anamnesis collection, the main objective of the visit was to detect possible contraindications for patient safety and therefore to understand if the condition was of their competencies (P1-P11).

In my assessment of the patient first there is the history and differential diagnosis, then I integrate the SD… they remain well separated moments for the patient's safety. (P3)

According to the clinical experience of osteopaths, there is a link between patient health processes and the presence of SD (P1-P11). Participants reported that SD is a potential index of AL for the patient. They also consider the related cumulative effects as contributing factors affecting the patient's ability to adapt to environmental challenges (P1, P4, P5, P6, P7, P9, P10). However, some participants specified that it was impossible to prove with certainty and rigor the influence of DS on the patient's health due to the poor research in this emergent field (P5, P10).

In general there can be a relationship between the presence of SD and the health of the patient…. sometimes SD does not only cause specific symptoms in the dysfunctional area, but creates an effect on the whole person: from a psychological point of view, of energy, but also alterations on other functions such as digestive, respiratory and cardiovascular. (P11)

Today we don't have evidence to demonstrate this kind of (SD-health) relationship. Clinical practice relies a lot on that, but we don't have the evidence to prove it. (P5)

Participants showed heterogeneity in the management of clinical uncertainty. It has been reported that SD was used as a simplifying tool for decision-making (P1, P3); theoretical models such as the biopsychosocial model (i.e. effective communication to improve therapeutic alliance), and to complexity medicine framework (i.e., cinefyn to address decision-making processes) (P4, P5, P9) were among the proposed strategies. Morevorer, an inductive clinical-diagnostic process, where experience, palpation, and treatment developments lead to an understanding of the complexity of the person emerged (P2, P7, P8, P10).

The SD represents topographically in the patient the point from which I have to start and input … so the SD represents a gateway to the complexity of the patient. (P3)

Theme 2: The treatment of SD needs to be shared within the healthcare team and the patients with the aim of improving their body awareness [see Additional file 2]

Analysis of the interviews revealed the importance of person-centered treatment, which aims to promote the patient's health. Moreover, participants reported the importance of making the patient active and participating, through sharing the therapeutic approach and using enactive strategies to improve body awareness.

As it was reported by P5 the patients are involved to improve their agency: *"It is necessary to make the patients understand that something is changing by looking at his daily life habits: how he walks, how he stands, how he washes, how he dresses, how his work is going or how better he can work."*

According to the participants, the therapeutic aim of OMT is to ensure the improvement of the patient's health status and adaptive capacity (P3, P6, P9, P10, P11). If SD shows a marked influence on the patient's functions and presentation, it would be a key point for the treatment; on the contrary, it would take less importance and wouldn’t be part of the treatment (P1, P3, P4, P7, P8, P10). The treatment is therefore structured around the person at the time of the consultation (P1, P4, P6, P7, P8, P9, P10).

The aim is always to go in a direction of salutogenesis … my work always depends on the person and the clinical moment. (P10)

Particular emphasis was placed on improving patient body awareness; not only possible through the specific effects of OMT, but also through enactive clinical strategies. This would not only increase engagement with the patient but could also play a useful placebo role in the success of the treatment (P1, P3, P5, P6, P8, P11).

For the patient this thing of the concordance between a perceived symptom and an area possibly involved with the perception of the symptom and the cessation of the symptom as a result of a technique, may have a certain kind of influence from a placebo point of view … the patient becomes aware of a present relationship between an area of the body, his\her possible symptom and the cessation of the pain sensation, which could the patient to have a different perception of his body and a different identification of his main symptom. (P1)

Different communication strategies emerged to explain the SD concept to the patient. The use metaphors, which have been transmitted over time by exponents of osteopathic practice (P1, P3, P6); A more immediate language, related to the patient's feelings, in order to create a direct bridge between the theoretical definition of SD and the patient's perception of it (P2, P4, P5, P7, P8, P10).

When I have to explain to the patient the concept of SD, I use my usual classical metaphors … then I explain that the osteopath assesses structure\function and treats to restore functions of the musculoskeletal, nervous, immune and endocrine systems. (P6)

According to participants, SD could be used as an interface of dialogue with other health professionals, in order to understand and define the positioning of osteopathy in the patient's care environment (P2, P3, P4, P5, P6, P9, P10). Furthermore, one of the main competencies of the osteopath represents the expert able to analyze, understand, integrate and treat the functional alterations of the patient in order to improve the body awareness and the health status of the person (P4, P9).

Medical specialists and other health professionals do their own examinations and detect altered function parameters that we could call SD as osteopaths, but then many times they don't know how to interpret what they find, so they identify me as the interpreter of the objective finding. What they want to know is why this thing is not functioning well and what is not working as it should. (P9)

*Theme 3:* The development of the SD concept in research and practice to better clarify osteopathic profession identity [see Additional file 2]

Osteopaths consider SD as one of the milestones of the osteopathic profession. SD is a fundamental element in the osteopathic health adaptive approach to the person, as osteopaths consider SD as related to AL sources, such as body functions impairments. However, participants also reported the need to expand our knowledge of SD.

In this regard, P7 claimed that *“It's now time for us to proceed along this scientific research road and we should go towards scientificity to be better recognised by the healthcare system".*

Participants expressed different views on the theoretical definition of SD describing it as a communicative key point between the practitioner and the patient (P1, P3, P8)or referring to the definition drafted in the glossary of osteopathic terminology (P5, P6, P7, P8, P9). Anyway, there is an emergent mindline in considering SD as a local adaptation syndrome (P2, P4, P6, P9).

SD is an alteration of the body structure, not only articular but also fascial and all related functions. It has an adaptive function to try to find the best possible conditions locally to allow the person to live his or her daily life. (P6)

Participants reported that SD is one of the specific elements that characterize the osteopathic identity and differentiates it from other health professions (P1, P3, P4, P5, P6, P8, P9, P10, P11). All participants stated that they consider SD as a fundamental part of their osteopathic education and clinical experience (P1-P11). Moreover, they pointed out that although SD is an important element for osteopathy, the community of practice is still debating about its usefulness in contemporary clinical practice (P1, P2, P3, P4, P5, P6, P10).

I integrate and treat SD in my treatment because I am an osteopath! (P3)

SD is a good working tool for osteopaths. Although not much is actually known about SD and research on it should be improved. (P10)

Participants reported the absolute importance of increasing their knowledge about SD characteristics and pathophysiological mechanisms (P2, P3, P4, P5 P6, P7, P10). The confusion on SD among the osteopathic clinical environment is also caused by the absence of well-structured scientific evidence (P2, P3, P4, P5 P6, P7, P10).

We don't really know all the pathophysiological mechanisms behind SD, and we don't yet have a fully effective reference model … we don't know exactly how SD works and there is a need for research in this direction. (P6)

Another key topic of discussion on scientific research was inter and intra-rater reliability. Participants expressed the need to improve reliability values through standardized palpatory training validated by research and using patient perceptual feedback. This would make the osteopath's palpatory findings possibly more reliable and in agreement with contemporary clinical practice requirements (P1, P2, P4, P5). However, it has been emphasized that SD is an ontologically subjective finding which characterizes the personal aspect of osteopathic treatment (P2, P3, P7).

I am not worried about the lack of intra-operator and inter-operator reliability. If the SD becomes an entity to interact with the system-patient, the SD would acquire a typically subjective value. Subjective from the point of view of the operator, the patient and the new entity (operator-patient relationship) that is created. (P3)

## Discussion

The present qualitative study investigated the attitudes and preferences about the use of SD in osteopathic patient care. The main conceptual framework of the study allows the researchers to evaluate narrative resources of life experiences and to present constructivist perspective in themes to describe pre-given essences and structures of experienced osteopaths. It explores the lived experiences of clinical role and relevance of SD. Despite the debate within the profession highlighting critical points regarding the validity of the concept of SD [13, 24, 28], a recently published scoping review confirms that SD is considered a commonly addressed entity in osteopathic practice [28]. The authors [28] analyzed 280 studies and discussed the role of SD in the osteopathic field, including information about assessment, modalities and time frame of treatment, and professional characteristics. The present thematic analysis followed the call for future qualitative studies suggested in the conclusions of the scoping review [28] to highlight osteopaths’ attitudes and preferences during clinical practice. It allows achieving another step in developing renovated osteopathic care theoretical models, bridging the potential gap between the use of SD in the osteopathic practice and the notion reported in the research field.

**Fig. 3:**
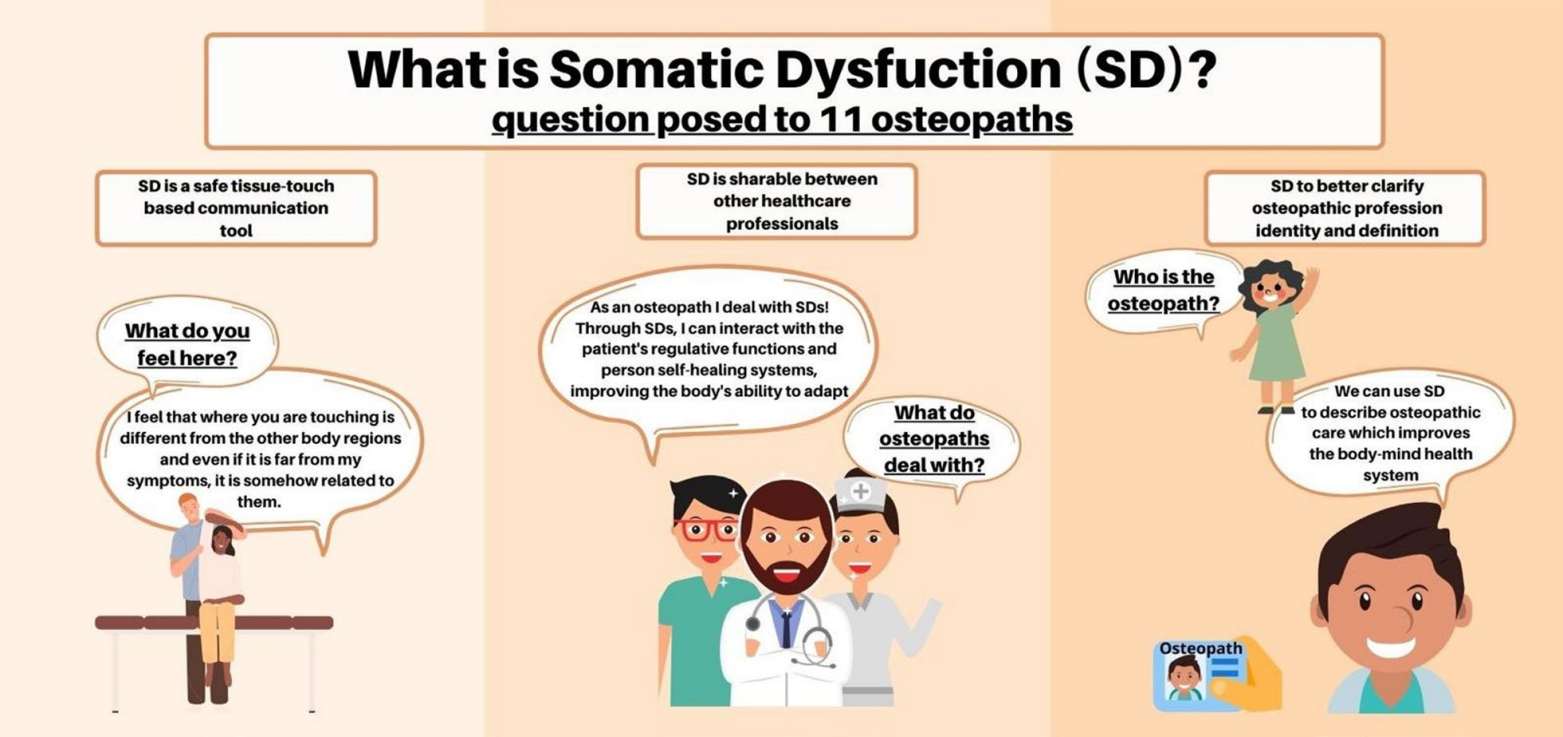
Somatic dysfunction according to the point of view of the participants

The following paragraph will discuss the most significant findings following the three main themes that arose from data analysis (Fig. 3).

The first theme emerging from the data analysis has highlighted participants' idea of SD as a safe tissue-touch based communication tool between operator and person complex adaptive health system. Participants refer to SD as an outcome of a touch-based participative interactive process between osteopath and patient, also shared by verbal communication respectful of the patients' expectations. The respondents' attitudes are to consider SD as an emergent pattern of body framework and systems interdependence that informs the sense-making of the different complexity domains of the daily clinical scenarios [48]. Furthermore, the role of SD in osteopathic diagnostic-clinical reasoning is mainly to address the touch input quality in the region of the entire body to improve biological and psychological self-regulation, focusing on patient agency, body awareness, and adaptive capacity. The interviewees reported 3 phases that characterize the decision-making process, in temporal order:

1. Assessment of contraindications to osteopathic treatment to ensure patient safety; confirming the findings of another qualitative study [53]. However, a recently published systematic review reported that further well conducted studies are needed to confirm and extend the safety of osteopathic care, despite no serious adverse events have been reported in the selected studies [54].
2. SDs assessment and their relationships with the patient's functional alterations through palpatory tests. This phase, in which the practitioner operates palpation and passive/active movements to perform provocation-inhibition tests [55–57], is considered by the participants as a clinical experience-dependent tool, but important to identify local and global functional alterations;
3. Identification of the SD’s relevance for the patient's presentation and needs. This is partially conferred by the severity of the SD (considered by the presence of one or more clinical signs expressed by TART) but is effectively validated by the link between SD-functional alterations and patient feedback. In other terms, it is evaluated in a shared decision-making process if a specific type of touch, executed in a particular area, can evoke perceived changes in patient agency, in the ability to perform daily movements, or perform a specific objective examination test. During the assessment of SD, an area of interest for both patient and osteopath, practitioners investigate patients' body awareness to better understand their psychological perceptions in exploratory verbal and non-verbal communication [58]. OMT application to distal body areas apparently not connected with patient complaints, but perceived as linked with the symptomatic region by the patient, could be seen as a body awareness improvement strategy [7].

The mentioned approach is not dissimilar from the most widely utilized method to justify a treatment application site used by chiropractors: i.e., Pain, Asymmetry, relative Range of motion, changes in Tissue temperature/texture/tone, and findings from Special tests (PARTS) [3]. These constructs define somatotopic relationships between the patient’s perceptions, signs, symptoms, and biological substrates. The methods to assess different items can not be considered without the patient's involvement [3]. The physical examination should be contextualized by the patient’s history and presenting complaint to progressively narrow the focus of attention, first to the region then the local site, and, sometimes, tissue. Assessment methods that replicate the patient’s familiar symptoms may be the most consistent sources for diagnostic manual information [3].

The putative model for the selection of personalized osteopathic approaches [59, 60] can be seen in the light of the enactive–ecological model to guide patient-centered osteopathic care [61]. According to the enactive framework, the familiar symptoms can be considered useful tools for the expectation of pain (prior) to be confirmed by the movement (confirmation of prior) through the active inference [61]. Consequently, the palpatory findings, following osteopaths' intuition and manual assessment, can be confirmed or not by the patient's pleasant or unpleasant perception of the different types of touch in the body framework regions [59–61]. As an example, during the osteopathic assessment (i.e., provocation-inhibition tests), the expectation of pain (prior) could be violated by feeling no pain during the movement executed while the osteopathic personalized touch is applied to the region (related to SDs), then generating a high prediction error and a new prior [59–61]. These results are also in line with the studies that underline how the use of these parameters focused on patients’ perceptions would not only increase the reliability of palpatory findings [55, 56] but also their relevance in the clinical decision-making process [59, 62–65]. Moreover, the results of a recently published observational study [66] highlighted an active inference perspective to solve the somatic dysfunction conundrum [24]: osteopaths might consider to collaborate with patients during functional testing to facilitate the emergence of the best adaptive movement patterns that better fit with the patient’s needs. Contextually improving awareness about their pain during active motion and re-contextualising their sensory feedback and alter their generative model.

Finally, experienced osteopaths reported using empathic verbal communication with non-verbal interaction and proximity approaches based on touching body regions of interest for patients and osteopaths, such as SD [59, 60]. In this context, SD is considered a valuable tool to build a therapeutic alliance emphasizing the person's centrality in the entire clinical process [59–61]. Through the SD shared-assessment process, the patient becomes an active participant during the clinical encounter, especially improving the effective body awareness perceptions and all other managing systems involved in adaptivity to the environment, maintaining health, and managing illness [59, 60].

The second theme emergent from experienced osteopaths' perspectives describe that the treatment of SD needs to be shared within the healthcare team and the patients with the aim of improving their body awareness. According to the participants’ perspective, SD results in a potentially helpful concept for sharing the object of the osteopathic person-centered intervention with mainstream healthcare professionals. Thanks to the description given by the International Classification of Diseases (ICD, which reports the different locations of body regions SDs) [67], SD is included into a worldwide shared conceptual framework independent of language and culture [68]. Moreover, experienced osteopaths consider the focus of osteopathic person centered care on patients’ agency, adaptability, physiological and psychological functions related to the body framework (i.e., SD) as a sharable knowledge in the health care teams. Concerning the role of SD in clinical practice, participants of this study believe that SDs could be associated with AL and adaptive capacity, and patient health. Participants' explanation of SD is consensual with the definition reported in the glossary for osteopathic terminology [9] and informed by the renovated osteopathic models [60, 69]: it refers to an altered regulative function associated with related inflammatory signs palpable in the body framework in different body regions that can be remote from the symptomatic area and shared with the patient. Osteopathic touch is focused on SD of the musculoskeletal body framework interacting with self-regulation, including psychological adaptation to social context [28]. Furthermore, participants’ reports are in line with recent findings, where SD is related to patient adaptive pathways and could represent one of the secondary outcomes associated with AL processes [25]. This constitute a fundamental element in the osteopathic person-centered approach aimed to improve patients’ autonomy in health and illness conditions: promoting biological adaptability [70], i.e., movement variability [11], as well as psychological flexibility to the environment, i.e., body awareness [71], interoceptive accuracy and the sense of self [72–78] and brain functional connectivity [79].

The third theme emphasizes the need to develop the concept of SD in research and practice to better clarify the osteopathic professional identity. Concerning the role of SD for the osteopathic profession, expert osteopaths in this study considered SD as one of the few main points that characterize osteopathy, representing the evolution from a traditional concept to a renovated model informed by current knowledge. As discussed previously, participants clearly departed from the purely mechanistic and simplistic conception of SD, emphasizing the centrality of the person's perceptions in the entire clinical process. These results mark the holistic nature of the osteopathic approach, but at the same time requires a deeper understanding of the relationship between SD and the patient's health processes. These present study's findings are in line with the vision reported in a recently published professional commentary [80] and a narrative review [69]: the participant's point of view appeared to be quickly informed by the renovated concept for SD proposed in the decision-making radar plot and algorithm based on structure/function/environment models renovation for a person-centered osteopathic approach [69]

Participants consider SD as a gateway to the complexity of the patient [48]. A neuro-myofascial active area that might act as an osteopath-patient interface to transmit the biological and physiological effects of touch, as reported by Baroni and coauthors [59]. In the light of the ecologic-enactive perspective [61, 76, 78], SD could be considered participatory access for the osteopath-patient dyad. A clue to support adaptations and promote patients’ ability to regain their agency to experience their daily living actions, thus enhancing their health and well-being.

The finding of the present study could inform the stakeholders involved in the updating process for the glossary of osteopathic terminology [81], and in particular the definition of somatic dysfunction, that resulted more related with patient perceptions than in the classical description. There are future implications for education in which it must be considered the implementation of clinical training with real patients (and their perceptions) during peer practice.

In conclusion, this study reflects the common thought of professionals that in order to support the establishment and development of the osteopathic profession within the healthcare environment, it is necessary to corroborate all that specifically characterizes it. For this reason, it will be necessary to reinforce the concept of SD by building a solid shared framework validated by evidence.

To obtain a broader view of the phenomenon, further studies should be conducted to explore osteopathic physicians’ perspectives on the effectiveness construct and evaluate the extent to which their views are consistent or in conflict with those of the patients. The present qualitative study aimed to better clarify practitioners’ attitudes and beliefs toward manual assessment and palpatory findings, firstly in the osteopathic field. Nowadays there are available data to start updating the concept of somatic dysfunction and unravel what part of the community of practice is perceived as an enigmatic role or influence of the SD in osteopathic practice [13, 28]. However, there is a need to produce compelling evidence of the SD of biological and psychological compounds [24]. Future studies should aim to generate an initial outline of the shared model for the different manual therapies as already presented in Castagna et al. study [60]. A further step might be represented by experimental studies to clinically test the generated model. Once a body of literature will be generated, there will be a need for dissemination among educational providers and professionals. It will implement a more consistent model in clinical practice.

To the best of our knowledge, the present thematic analysis is one of the few qualitative studies conducted on the topic. We acknowledge some limitations, first the study was carried out only on Italian osteopaths. Moreover, only one of the participants was female [45, 82, 83]. Second, the findings may not be entirely able to generalize to other nations or circumstances, nor can they be used to characterize the entire osteopathic profession, indeed future studies including participants from other nationalities, and with a more balanced gender representation could generate data for an overall framework for the model [45, 82, 83]. Moreover, the participants were selected not just with purposive sampling but through referrals by other participants. This could have led to a polarization of the perspectives resulting in a possible selection bias. The above-mentioned limitation was mitigated by the clinical and academic experience of the participants in Italy as well as in other European countries, and by their different training and academic title achieved in Italy, France, Germany and United Kingdom.

Furthermore, despite the research team underwent a self-reflection activity to express their own personal beliefs prior to the data analysis, we cannot exclude that, especially where researchers had the same prior opinion on a specific topic, self-confirmation biases might have occurred in the interpretation of the interviews [43].

## Conclusions

A panel of expert Italian osteopaths consider the concept of SD as a valuable tool integrated into the osteopathic evaluation and treatment process. The shared concept and clinical application of SD is informed by person-centered care concepts and from the fields of neuroscience, cognitive and complexity science. Our study reports a common need among osteopaths to develop an evidence-based framework of SD to allow the best development of the osteopathic profession. Moreover, this study could help the scientific community in developing a uniform framework for the use of palpatory findings in manual therapies.

## Supplementary Information

**Additional file 1**: Semi-structured interview

**Additional file 2**: The most relevant participants’ quotes

## Data Availability

The data presented in this study are available on request from the corresponding author.

## Abbreviations

AL: Allostatic load
COREQ: Consolidated Criteria for Reporting Qualitative Research
ICD: International classification of diseases
OMT: Osteopathic manipulative treatment
PARTS: Pain, Asymmetry, relative Range of motion, changes in Tissue temperature/texture/tone, and findings from Special tests
ROI: Registro degli Osteopati d’Italia
SD: Somatic dysfunction
TART: Tissue texture alteration, positional Asymmetry, altered Range of motion, Tenderness
