# Supplementary material for: “What you feel under your hands”: exploring professionals’ perspective of somatic dysfunction in osteopathic clinical practice—a qualitative study
Source: Chiropr Man Therap. 2022 Aug 31;30:32. doi: 10.1186/s12998-022-00444-2 (PMC9429724; doi:10.1186/s12998-022-00444-2)
Supplement: Supplementary file 1 — Additional file 1: Semi-structured interview [file 12998_2022_444_MOESM1_ESM.docx]

**Additional file 1. Semi-structured interview**

| 1. Thank you for participating in the qualitative research project on the clinical preferences and attitudes of Italian osteopaths in the practical application of somatic dysfunction. I will ask you some general information about your osteopathic training and practice. What is your name? How old are you? Where and in which institute did you study osteopathy? Up to which academic level? How many years have you been working in the clinical environment? How many years of teaching? Do you have a particular clinical osteopathic approach? Do you have evaluative and/or technical preferences? 2. Can you give me a definition of somatic dysfunction? What kind of practical and clinical interpretation do you give to SD? 3. Do you include SD in your decision-making and evaluation process? 4. If not:   I. Why?  II. What clinical findings do you look for in the evaluation process?  III. What kind of osteopathic evaluation does he/she perform?  IV. Do you use a particular conceptual model found in literature?   1. If yes:   I. Why?  II. How and how much does it influence decision making?  III. Referring to your clinical experience, do you think there is a relationship between patient health and the presence of SD? Have you highlighted a particular case history?  IV. How do you integrate SD with your medical history?  V. Do you consider SD during the differential diagnosis process?  VI. How do you integrate SD into the osteopathic objective examination?  VII. What kind of evaluative procedure do you use?  VIII. What is your SD assessment about?  IX. Do you use any instruments?  X. How do you integrate SD into the complexity of the patient?  XI. Do you take the patient's perceptions and feelings into account during the SD assessment phase?  XII. How do you explain the concept and presence of SD to the patient?   1. Does he/she include SD during the patient's treatment phase? 2. If no:   I. Why?  II. How do you treat the patient?  III. What does the treatment focus on?  IV. Do you use a particular conceptual model found in literature?   1. If yes:   I. Why?  II. Do you consider SD as a reference point for treatment? If no, what is the treatment based on?  III. How and with which techniques do you treat SD?  IV. How and in what way is SD important for successful treatment?  V. Do you take the patient's perceptions and feelings into account during the SD treatment phase?  VI. How do you evaluate the progress of SD in the context of clinical treatment?   1. Do you want to add something to the interview? Do you have something to ask me? What do you think about this interview |
| --- |
|  |
|  |
|  |
|  |
|  |
|  |
|  |
|  |
