# Supplementary material for: “What you feel under your hands”: exploring professionals’ perspective of somatic dysfunction in osteopathic clinical practice—a qualitative study
Source: Chiropr Man Therap. 2022 Aug 31;30:32. doi: 10.1186/s12998-022-00444-2 (PMC9429724; doi:10.1186/s12998-022-00444-2)
Supplement: Supplementary file 2 — Additional file 2: The most relevant participants’ quotes [file 12998_2022_444_MOESM2_ESM.docx]

**Additional file 2. The most relevant participants’ quotes**

| ***Theme 1:***  **SD as a safe tissue-touch based communication tool between**  **operator and person complex adaptive health system** | |
| --- | --- |
| **Assessment of DS in the person as a whole** | *"SD can be somehow parameterized or otherwise quantified through parameters defined by the TART acronym". (P4).*  *"The parameter that osteopaths objectively use the most is mobility and movement especially in the first degrees." (P6).*  *"I integrate SD into the objective examination through the application of structure-function correlation tests." (P2).*  *"I only take SD into account if clinically reflects the expression of an altered functioning of the patient, otherwise it is negligible ... it must always be placed in a context of globality." (P4).*  *"It is the agreement between my perception and the patient's perception that establishes the meaning of SD.... SD takes validity only when it is corroborated by the patient's altered perception and if it is in any way related to the reason for the consultation". (P1).*  *"Intra-operative palpation doesn't have that much reliability and reproducibility. Therefore I have a tendency to rely relatively only on my own hands and I believe that the reliability of palpation can be increased by perceptual feedback from the patient." (P1).*  *"First I identify the SD, then depending on the regional and systemic tests that I do, I try to understand and identify a series of connections between the various dysfunctional areas of the patient." (P10).* |
| **Operator communication through the tissues** | *"Dialogue through tissues is the only way I have found so far with my hands to change the functional parameters of the patient." (P2).*  *"The best technique for a specific SD you can feel it in the moment. It can't be categorised. It depends a lot on what you feel under your hands." (P7).*  *"I like talking to tissues, so even when I do an HVLA I always have a focus on what the tissues are telling me. We have to give the body the opportunity to tell us in real time how much and in what way our inputs are going well or not." (P10).*  *"In assessment and treatment there is a constant exchange of information between the hands and the body that tells you the type of approach to be applied, the force to be applied and the direction to be taken... the ability to have a continuous dialogue with the tissue then translates into the effectiveness of the treatment". (P11).* |
| **Patient safety** | *"In my assessment of the patient first there is the history and differential diagnosis, then I integrate the SD... they remain well separated moments for the patient's safety." (P3).*  *"First of all anamnesis and exclusion of red flags. I look later for SD. History and differential diagnosis process is always necessary first, and these are the priority in the evaluation of the patient". (P7).*  *"First I make an accurate diagnosis, only then do I take care to assess well those parts of the body that are related to the reason for the consultation and the SDs. Then I integrate the findings with the rest of the body". (P8).*  *"I first do a recent and past medical history analysis, then I do a differential clinical reasoning. Then when I go for palpation tests and I detect SD, then I try to correlate it with what the patient told me in the history and maybe I ask them other questions to understand better." (P9).* |
| **SD as an expression of allostatic load and the link with patient health** | *"There is a correlation between SD and health ... SD leads to impairment of function and this creates alterations in the health of the patient." (P9).*  *"Today we don't have evidence to demonstrate this kind of (SD-health) relationship. Clinical practice relies a lot on that, but we don't have the evidence to prove it." (P5).*  *"A SD, even if it does not give reported or appreciable clinical signs, when associated with other dysfunctions in other parts of the body, may already represent a potential reducer of the patient's ability to self-heal." (P4).*  *"SD impact on the subject's adaptability. This means that when the subject's ability to adapt is exhausted, then it can cause symptoms at that point". (P1).*  *"If we mean SD as alterations that require the body to demand adaptation, then SD can affect the patient's health: because they cause and create allostatic load." (P1)*  *"SD may reflect an alteration in health in the presence or even in the absence of pathophysiological conditions." (P2).*  *"I think that in general there is a relationship between patient health and DS. It depends from person to person.” (P6).*  *"SD is one of the possible alterations of function, not so much of the body structure, but for the global adaptive capacity of the patient ... SD may be detected in a specific area, but it reflects more systemic alterations and reduction of adaptive capacity." (P10).*  *"In general there can be a relationship between the presence of SD and the health of the patient.... sometimes SD does not only cause specific symptoms in the dysfunctional area, but creates an effect on the whole person: from a psychological point of view, of energy, but also alterations on other functions such as digestive, respiratory and cardiovascular". (P11).* |
| **SD management in patient complexity** | *"The SD represents topographically in the patient the point from which I have to start and input ... so the SD represents a gateway to the complexity of the patient." (P3).*  *"To help me with complexity I use the complexity scheme published by Lunghi and Baroni on the cynefin framework in osteopathy." (P4).*  *"In a situation of complex patients, I try to make that complexity simple starting from the most global assessment to get to the smallest ... it is the body that makes you understand and answers your questions." (P2).*  *"The complexity of the patient is the holistic aspect of the person. The bio-psycho-social context goes some way to address this complexity, in the way that the person is not just made of body, so the psychological and social aspect also have a role, as does biological complexity itself". (P5).*  *"In order to be able to approach the complexity of the patient I simply have to make sure that with my technique and my treatment, regardless of where I position myself, I can integrate into the patient's globality". (P8).*  *"Purely rational reasoning is wonderful but it doesn't work in practice and that's not how the body works. 1+1 does not always make 2. So it does not make sense to enclose the complexity of the person in linear reasoning. That is why I integrate SD into the decision-making process but do not always consider it important and central to the treatment."  (P10).* |

| ***Theme 2:***  **The treatment of SD is shareable between osteopaths, other health professionals, and the patients involved in the therapeutic pathway improving body-awareness and health** | |
| --- | --- |
| **SD and person-centered treatment** | *"Treatment is strongly influenced by the patient's ability and willingness at that time of the clinical encounter to receive one treatment rather than another… and also, by the whole previous specific and global assessment phase." (P4).*  *"The aim is always to go in a direction of salutogenesis ... my work always depends on the person and the clinical moment." (P10).*  *"The link between SD and patients is an important sign ... if I don't identify any link between what I found as SD and the patient, then I treat the person according to what I think is most appropriate for the patient at that time, but I will not address the treatment focus on SD." (P4).*  *"There are cases in which SD is not central to the treatment. For example, a person comes to me who has been suffering from back pain, but in the evaluation you don't find anything particular SD. While in the analysis of the person, it turns out that he/she has a very high level of anxiety and psychosocial distress, in my opinion it doesn't make much sense to start working in this case on the single SD ... the choice of the technique is not made on the basis of my capacities or my needs … It's selected on the basis of the patient's characteristics. So I have to do the best for the patient". (P6).*  *"I don't think SD is the focus of treatment. Treatment is first and foremost about listening and understanding where and how the patient's issues arise ... every somatic dysfunction presents itself in a billion different ways depending on the patient." (P7).*  *"Treating SD is important, but it is not essential. So SD has to be managed, it doesn't always have to be taken away ... Our job in any case is to try to get the patient back into a beneficial allostatic regime." (P9).* |
| **SD, body awareness and placebo** | *"It is necessary to make the patients understand that something is changing by looking at his daily life habits: how he walks, how he stands, how he washes, how he dresses, how his work is going or how better he can work." (P5).*  *"For the patient this thing of the concordance between a perceived symptom and an area possibly involved with the perception of the symptom and the cessation of the symptom as a result of a technique, may have a certain kind of influence from a placebo point of view ... the patient becomes aware of a present relationship between an area of the body, his\her possible symptom and the cessation of the pain sensation, which could the patient to have a different perception of his body and a different identification of his main symptom." (P1).*  *"If the patients come in having pain while walking or standing, I keep these things as a reference to see if anything has changed after treatment. Or I use the pain evoked in orthopaedic and osteopathic tests as a reference. But this is more a useful index for the patient to make him feel that something has changed". (P8).*  *"I try to make the patient understand the way he is feeling to make him understand that his symptom is the result of different functional and/or structural alterations." (P11).* |
| **Explaining the SD concept to the patient** | *"I explain SD  to the patient with the metaphor of the little donkey who can carry the weight better as we remove unnecessary bits." (P3).*  *"I don't talk much about SD with patients. If they ask me I tell them it's the stuff osteopaths deal with. I use metaphors like the glass of water." (P1).*  *"When I have to explain to the patient the concept of SD, I use my usual classical metaphors ... then I explain that the osteopath assesses structure\function and treats to restore functions of the musculoskeletal, nervous, immune and endocrine systems." (P6).*  *"When I have to explain SD to the patient, I make up some metaphors ... I try to make people understand rather than explain." (P7).*  *"I explain it as simply as I can ... I explain that there are parts of your body that don't move as well as they should and could affect the overall functioning of the body organism ... with the treatment I will try to release these parts so that then you can function better and have better functionality in your daily life." (P10).* |
| **SD in the interdisciplinary context** | *“Medical specialists and other health professionals do their own examinations and detect altered function parameters that we could call SD as osteopaths, but then many times they don't know how to interpret what they find, so they identify me as the interpreter of the objective finding. What they want to know is why this thing is not functioning well and what is not working as it should". (P9).*  *"In an interdisciplinary perspective, I would use SD to make others understand what we do, how we reason, that we are a ground medicine, and that SD is one of the specific osteopathic entities that allow us to interact with the patient 'ground'." (P3).*  *"It would be necessary to create a dialogue through informative conferences of all the health figures with osteopathy and to make them understand the intelligibility of what osteopathy can do in relation to the other health functions". (P2).*  *"Through the evaluation and treatment of SD the osteopath will be able to give his professional contribution within the healthcare team". (P4).* |

| ***Theme 3:***  **The development of the SD concept in research and practice**  **to better clarify osteopathic profession identity and definition.** | |
| --- | --- |
| **SD definition** | *"SD represents an entry point and an interface, where I can interact with the patient system ... it is a space where I meet with a patient." (P3).*  *"Regarding the definition of SD, I refer to the glossary of osteopathic terminology. So SD is an expression of impaired or altered function of a somatic component related to its myofascial, vascular and neural components." (P9).*  *"The SD is a neurologically active area of the patient to interface with the patient's central nervous system." (P1).*  *"The SD is the body's manifestation to a poor health adaptivity." (P2).*  *"SD can be defined as a functional alteration located in a specific body district”. (P4).*  *"The only definition we have is the one that is written in the glossary, so a purely osteopathic definition." (P5).*  *"SD is an alteration of the body structure, not only articular but also fascial and all related functions. It has an adaptive function to try to find the best possible conditions locally to allow the person to live his or her daily life." (P6).*  *"SD can be defined as a segment that not only compromises somatic function but also carries with it vascular, nerve, lymphatic, ligamentous and capsular functional components." (P7).*  *“The SD definition is related to the TART acronym. So difference in tissue texture, difference in vascularity, and not just at the biomechanical level of course, but all the components that go into making sure that somatic function is not preserved as it should be. So also the fascial, neural and circulatory part." (P10).* |
| **SD and osteopathic identity** | *"Today ‘somatic dysfunction’ is an osteopathic term that is still being debated within the practice community. So if we, as osteopaths, do not yet have a clear idea on SD... consequently it becomes difficult to communicate it to other practitioners." (P5).*  *"SD is one of the few important fixed points that osteopathy has to define its profession as unique and different from others." (P10).*  *"I include SD in the evaluation for the professional profile which has just come out and it turns out to be our main characteristic and which distinguishes us from all the other health professions and also from the other manual therapy professions because we are the only ones who have been electively assigned the normalisation of somatic dysfunctions". (P1).*  *"I integrate and treat SD in my treatment because I am an osteopath!" (P3).*  *"I definitely consider SD as a reference point, also because after the treatment I always go and retest it." (P4).*  *"If we take away the somatic dysfunction the only thing that characterises osteopathy a little bit goes away." (P6).*  *"I include SD in my osteopathic practice because it is a part of the whole that makes up the person. So if SD is present it means that there is no better adaptation for the body. (P8).*  *"Well I include SD in the assessment of the patient because the osteopath deals with SD, so I don't see why I shouldn't include the assessment of SD. In my opinion, it is the first thing the osteopath has to do, beyond what the patient has. My job is to find SDs with functional tests and then go and correct them." (P9).*  *SD is a good working tool for osteopaths. Although not much is actually known about SD and research on it should be improved." (P10).*  *"SD is confusing to us osteopaths so even more confusing is to the patients." (P1).* |
| **SD and scientific research** | *"We don't really know all the pathophysiological mechanisms behind SD, and we don't yet have a fully effective reference model ... we don't know exactly how SD works and there is a need for research in this direction". (P6).*  *“I am a little perplexed by people who are beginning to totally deny the existence of SD... perhaps we do not know all the physiopathological mechanisms behind it ... but totally denying it seems illogical to me, rather let's say that we do not know its characteristics in full". (P6).*  *"To better understand the nature and influence of SD on the patient, it is necessary to conduct clinical trials that aim to understand SD in whole body, loco-regional or segmental assessment. In addition, the treatment performed must be protocolized or at least black box." (P4).*  *"In my opinion, we should do a lot of research to understand how the central nervous system, the autonomic system and the whole PNEI axis reacts when we touch people.” (P10).*  *"Only after reviewing the complex concept of DS can one think of assessing reliability through scientific studies." (P4).*  *“In researching the understanding of SD it is necessary to detect: tissue densitometric parameters, or pre- and post-treatment skin and subcutaneous haematochemical parameters in areas where SD has been diagnosed.” (P2).*  *“Another important aspect in the development of SD research concerns the neurophysiological aspects of SD: something has been done to repeat the studies of Denslow and Korr, and I would like to see more of them using the maximum technological capacity available. Collaboration in research with physiologists is important in this respect.” (P3).*  *“Scientific research on DS is lagging behind for several reasons; one reason is because osteopathy has always somewhat rejected evidence-based practice. So it is lagging behind other professions.” (P5).*  *“It's now time for us to proceed along this scientific research road and we should go towards scientificity to be better recognised by the healthcare system". (P7).*  *"By using motor variability in conjunction with the concept of TART we can increase the reliability of the clinical sign of SD. This is fundamental, because it means making an assessment that is in some way repeatable within the same operator and between different operators. Consequently, it is more objective as data." (P5).*  *"I am not worried about the lack of intra- and inter-operator reliability. If the SD becomes an entity to interact with the system-patient, the SD would acquire a typically subjective value. Subjective from the point of view of the operator, the patient and the new entity (operator-patient relationship) that is created." (P3).* |
